# Ecological factors associated with suicide mortality among non-Hispanic whites

**DOI:** 10.1186/s12889-020-09379-w

**Published:** 2020-09-03

**Authors:** Nick Graetz, Samuel H. Preston, Morgan Peele, Irma T. Elo

**Affiliations:** Department of Sociology and Population Studies Center, University of Pennsylvania, 3718 Locust Walk, Philadelphia, PA 19104 USA

**Keywords:** Suicide, Non-Hispanic white, Opioids, Religion, Manufacturing, Gun control, Education, Marriage

## Abstract

**Background:**

In this paper, we examine the ecological factors associated with death rates from suicide in the United States in 1999 and 2017, a period when suicide mortality increased in the United States. We focus on Non-Hispanic Whites, who experienced the largest increase in suicide mortality. We ask whether variation in suicide mortality among commuting zones can be explained by measures of the social and economic environment and access to lethal means used to kill oneself in one’s area of residence.

**Methods:**

We use vital statistics data on deaths and Census Bureau population estimates and define area of residence as one of 704 commuting zones. We estimate separate models for men and women at ages 20–64 and 65 and above. We measure economic environment by percent of the workforce in manufacturing and the unemployment rate and social environment by marital status, educational attainment, and religious participation. We use gun sellers and opioid prescriptions as measures of access to lethal means.

**Results:**

We find that the strongest contextual predictors of higher suicide mortality are lower rates of manufacturing employment and higher rates of opiate prescriptions for all age/sex groups, increased gun accessibility for men, and religious participation for older people.

**Conclusions:**

Socioeconomic characteristic and access to lethal means explain much of the variation in suicide mortality rates across commuting zones, but do not account for the pervasive national-level increase in suicide mortality between 1999 and 2017.

## Background

Roughly 800,000 deaths from suicide occur every year globally [56]. In the United States, 47,000 deaths from suicide were recorded in 2017 [31]. Between 1999 and 2017, the age-adjusted suicide rate rose 33% from 10.5 per 100,000 to 14.0 per 100,000. Females exhibit a sharp peak in suicide rates at ages 45–64 while rates for males are relatively flat across the working ages and then show a large increase at ages 75+ (Ibid.). For Americans 40 years and younger, suicide deaths are only exceeded by motor vehicle fatalities [32].

Suicide is often understood as an intensely private and personal act, with the focus of analysis on the mental and emotional health of an individual [37]. While most individuals who die by suicide have a history of mental illness, many of the circumstances and conditions that precipitate suicide are properties not of individuals themselves but of relations between individuals and groups [57]. Durkheim [23] proposed that a key factor in suicide risk was one’s degree of social integration- the sense of social belonging and inclusion, the love, care, and concern that can flow from social ties [57]. Durkheim’s profound insights about the centrality of social relations to human well-being have been supported by evidence that the evolution of the human brain was driven by the advantages larger brains offered for engaging in complex social relationships [24].

In this paper, we examine the relationship between suicide mortality and features of social relations in one’s area of residence that either may increase or mitigate against individuals’ vulnerability to suicide. One of the main features considered relates to the availability of jobs in an area, reflecting the role that individuals play in creating products and services of use to themselves and to others. Lower-ranking occupations are associated with above-average mortality from suicide [9, 18, 57]. Job loss in a modern economy may increase mental distress and the subsequent risk of suicide by virtue of a loss of personal identity and of the means of financially contributing to family and the larger group. Indeed, studies have found that unemployed individuals in the United States have sharply elevated suicide death rates, underscoring employment opportunity as a key social determinant of suicide risk [22, 38].

### The decline in manufacturing

We pay special attention to the availability of manufacturing jobs in an area because of recent massive declines in manufacturing jobs in response to both rising import competition and capital intensification of methods of production [16]. Manufacturing is an “export” industry in the sense that most of the product is consumed beyond the local area where it is produced, in contrast to much of the product of service industries. Unless declines in manufacturing are replaced by increases in other export industries, local areas may be especially hard hit. Manufacturing is also highly concentrated spatially. Case and Deaton [13, 14] have proposed a link between increasingly adverse labor market conditions in the U.S. and rising mortality from suicide. They extend their hypothesis to mortality from alcoholic liver disease/cirrhosis and drug overdose as well, combining these causes of death under the rubric of “deaths of despair”. Their analysis uses aggregate time series data and does not investigate spatial relations between labor markets and mortality from deaths of despair (Ibid.).

It is possible that the decline in manufacturing jobs has been especially demoralizing for men, who predominate among employees in the industry. In 2017, 70.5% of workers in manufacturing were male [53]. The erosion of earnings opportunities for men threatened the traditional breadwinner/homemaker family that had evolved over centuries and that remains a powerful organizing ideal [21, 45]. Under this model, men fulfill their gendered responsibilities to the family primarily through productive activities outside the home [10, 29]. The decline in manufacturing (as well as in mining and farming, also highly masculine industries) undercut the “gains from trade” resulting from complementarities between what men and women brought to marriage [5]. Women were affected as well. The decline in male jobs often encouraged their spouses to enter the labor force [28], adding a “second shift” on top of their childcare and housework duties [34]. Meanwhile, women’s own employment was sometimes threatened by men who had lost jobs and entered traditionally female occupations and industries [28]. Indeed, a wide array of powerful ethnographic accounts attests to the demoralization of both men and women and their communities that has accompanied the loss of manufacturing jobs [33, 49, 54, 58]. For example, Silva [49] highlights the lives of men and women struggling to cope with feelings of anxiety and depression in a declining coal town. Residents of the town describe suicide as “taking the easier route” compared to facing reality head-on and pushing through their hardships ([49]: 75). Some individuals resort to firearms, while others “pray that God will take them out of life” ([49]: 161).

### Other ecological factors

Beyond characteristics of the labor force, we introduce data on five other contextual-level characteristics hypothesized to affect suicide death rates. Two other features of social relations have figured prominently in explanations of variation in suicide mortality, including those of Durkheim: family circumstances and religious participation. Both family and religious groups offer the possibility of connectedness in stable, durable relationships. Consistent with this expectation, married individuals have lower suicide rates than the unmarried [22, 38]. Trgovac et al. [52] find that counties with high proportions separated and divorced had higher suicide mortality among working-age men during 2000–06. Others have found a link between marriage and lack of well-paying jobs especially in regions where automation and trade have reduced access to such jobs [3]. A multivariate analysis of the 1993 National Mortality Followback Survey concluded that participation in religious activities reduces the odds of suicide occurrence [42].

High levels of education may reduce the risk of suicide by providing individuals with higher incomes that reduce stress from their social environments and provide better skills for managing the stresses that exist [22, 36]. High levels of educational attainment in an area may be associated with the provision of superior social services including suicide prevention activities. At the individual level, people with low levels of schooling have higher suicide rates [22], and the recent increase in suicide mortality among non-Hispanic Whites is most evident among those with less than a Bachelor’s degree [12]. One previous study found that the inverse relation between schooling and suicide risk was limited to males [38].

While our analysis focuses on possible vulnerabilities to attempting suicide, it is necessary to recognize that access to lethal means to kill oneself is also likely to affect death rates. In the U.S., the case fatality ratio for suicide attempts is strongly related to the availability of household firearms [41]. On the individual level, having a firearm in the home has been linked to an increased risk of suicide [41]. On the state-level, rates of suicide death are lower in states with more gun control policies, with several studies finding more background checks to be associated with lower risk of death from suicide [1, 27, 35, 48]. States with a higher prevalence of gun ownership have been found to have higher rates of death from suicide and from suicide by a firearm [43].

A final variable reflects the possible role of medical and pharmaceutical activities in suicide patterns. The dramatic expansion of opiate prescriptions during the twenty-first century may have had the effect of reducing suicide mortality by virtue of reductions in the prevalence of severe pain. On the other hand, the widespread availability of opiates provides a lethal means that is readily at hand for those who are motivated to attempt suicide. And opiates may also distort mental processes in ways that lead to self-destructive behavior. Although the number of suicides due to drug poisoning increased somewhat over time, the percentage of suicides at ages 20 and above attributed to drug poisoning remained relatively stable between 2000 and 2017. It is possible that suicides from drug overdose are underestimated due to the difficulty of ascertaining intent [44].

### Issues of research design

Although the basic question we ask is best addressed by a combination of individual and aggregate-level data, this option is not available at present and, like many studies of suicide, we use aggregate data. Inferring causal connections between employment conditions and suicide from individual-level correlations alone is hazardous. Statistical confounding and reverse causation are serious threats to unbiased estimation. Depression, anxiety, and other debilitating mental conditions can affect one’s employment conditions as well as the likelihood of suicide [19]. Opioid use can simultaneously affect one’s mental health and one’s employment circumstances [16, 46]. Because of these potential biases, we measure employment conditions at an aggregated rather than an individual level in order to be able to treat them as exogenous to the individual. This approach has also been employed to investigate the impact of employment conditions on mortality from drug overdose [16, 46].

Because the sharp rise in suicide rates during the twenty-first century coincided with a rapid decline in the manufacturing workforce, we chose a research design that would enable us to investigate the relationship between these two trends. Wherever possible, we measured our variables, including suicide mortality, at two widely-separated periods during the twenty-first century: 1999–2001 and 2015–17. Our analytic plan combines data from the two periods and includes a variable indicating the date of observation. In this way, we estimate the national-level average change in suicide mortality conditional on time-varying contextual characteristics of commuting zones and examine whether this can be accounted for by shifts in these local variables.

In both 2015 and 2016, the non-Hispanic white age-adjusted suicide death rate was nearly three times the non-Hispanic black rate and 2.5 times the rate for the Hispanic population [59]. Case and Deaton’s [12–14] analyses of rising mortality from deaths of despair were focused on Non-Hispanic Whites. Because of the large racial and ethnic disparities in levels and trends in suicide mortality, we have limited our analyses to Non-Hispanic Whites. As noted earlier, the age-patterns of suicide mortality are quite different for men and women. These differences suggest that there may be interactions between our explanatory variables and age and sex. Accordingly, we estimate two sets of regressions for each sex, one limited to the working ages 20–64 and the other pertaining to ages 65+. We control for five-year age groups in each of these regressions.

## Methods

### Commuting zones

The local labor market we analyze is the commuting zone (CZ). These are relatively self-contained areas where the vast majority of residents also work. Unlike metropolitan areas, commuting zones span the entire U.S. [16]. The concept of commuting zones was developed by Tolbert and Sizer [51], who used county-level commuting data from the 1990 Census data to create 704 clusters of counties that are characterized by strong commuting ties within CZs, and weak commuting ties across CZs [2].

### Mortality data

We use restricted vital statistics data on deaths by age, sex, year, race/ethnicity, county and cause of death available from the National Center for Health Statistics for each U.S. county under a data user agreement. We use public-use Census bridged-race population estimates by age, sex, county, and year to calculate age-specific death rates. The county-level data are aggregated to the 704 commuting zones covering the continental United States (704 out of the total 709 defined by the USDA in 2000). In each commuting zone we collapse deaths and populations to two pooled years (1999–2001 and 2015–2017), sex, and 5-year age groups. Deaths from suicide are based on the ICD-10 codes X60-X84 and Y87.0. As described below, we also included Y10-Y34 (deaths of undetermined intent) in sensitivity analyses.

### Contextual characteristics

Our contextual-level variables include two measures of economic conditions, percent of the labor force in manufacturing industries and percent of the labor force who are unemployed (Supplementary Table 1). We also include percent of the sex-specific Non-Hispanic White population ages 25 and above with a college degree, percent of the population ages 15 and above who are married as a measure of family structure, percent of the total population with religious membership, and annual opioid prescriptions per 100,000 population. In our main analyses, we include gun dealers per capita as our measure of firearm availability, which is measured at the level of the commuting zone. We also examined two alternative measures of firearm availability: percent of households in the state with a gun and a score of state restrictions on gun ownership based on Rand State Firearm Law Database. Both measures were associated with suicide mortality (results available from the authors upon request). All contextual variables are standardized across both periods (mean = 0 and standard deviation = 1), such that coefficient estimates are associated with a 1 standard deviation increase in the contextual variable relative to the national average across both periods.

### Suicide mortality models

The following negative-binomial model was estimated separately for males and females and for ages 20–64 vs. 65 and above:

1 $$ {D}_{c,y,a}= NB\left({m}_{c,y,a}\ast {P}_{c,y,a},\theta \right) $$

2 $$ \log \left({m}_{c,y,a}\right)={\beta}_0+{\boldsymbol{\beta}}_{\mathbf{1}}\ast {\boldsymbol{A}}_a+{\beta}_2\ast {Y}_y+{\boldsymbol{\beta}}_{\mathbf{3}}\ast {\boldsymbol{X}}_{c,y,a}+{\varepsilon}_{c,y,a} $$

Where *c*, *y*, *a* are the indices for commuting zone, pooled year (1999–2001, 2015–2017), and 5-year age group; *θ* is the overdispersion parameter of the negative-binomial distribution; *D*_*c*, *y*, *a*_ and *P*_*c*, *y*, *a*_ are the counts of suicide deaths and population, respectively; *m*_*c*, *y*, *a*_ is the underlying mortality rate from suicide in commuting zone *c*, pooled year *y*, and age group *a*; *β*_0_ is the intercept; ***A***_*a*_ is a vector of age group dummies and ***β***_**1**_ is the associated vector of regression coefficients; *Y*_*y*_ is a dummy for pooled year and *β*_2_ is the associated regression coefficient; and ***X***_*c*, *y*, *a*_ is a vector of contextual covariates for commuting zone *c* and pooled year *y*, and ***β***_**3**_ is the associated vector of regression coefficients. The negative-binomial model is a flexible alternative to the Poisson model for discrete count data when the sample variance may exceed the sample mean (i.e. the data are “over-dispersed”), as is the case with the present suicide mortality data. The single-parameter Poisson model implies that the mean and variance are equal, whereas the additional parameter defining the negative-binomial distribution, *θ*, can be used to adjust the variance independently of the mean (i.e. adjust for over-dispersion). We report a test statistic for the presence of over-dispersion in these data described by Cameron & Trivedi [8] and implemented using the *AER* package in R.

## Results

**Fig. 1:**
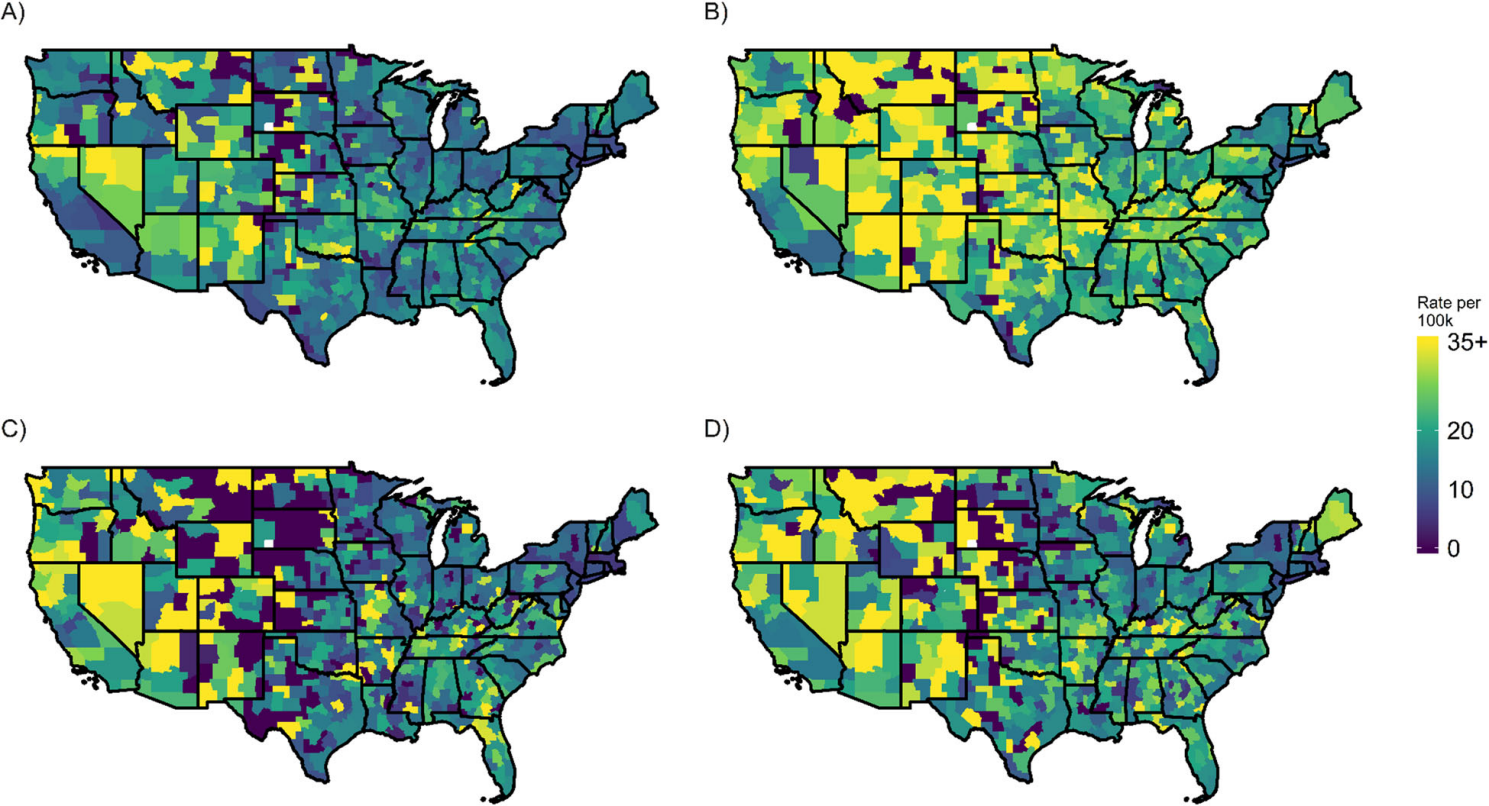
Age-standardized rates of suicide mortality (both-sex) by (**a**) commuting zones for ages 20–64 in 1999–2001 and (**b**) 2015–2017, and (**c**) ages 65+ in 1999–2001 and (**d**) 2015–2017. All shapefiles were obtained from publicly available Census data using the *tigris* R package [55]

Table 1 shows a sharp increase in suicide mortality for Non-Hispanic Whites between 1999 and 2001 and 2015–17. Male mortality rose by 36% at ages 20–64 and by 31% at ages 65+; female mortality rose by 80% at ages 20–64 and by 63% at ages 65+. The spatial pattern of suicide mortality at ages 20–64 in the 704 commuting zones between 1999 and 2001 and 2015–2017 is shown in Fig. 1 (age-standardized by 5-year age groups using the 2017 Census population estimates). In general, the eastern half of the country has lower rates than the western half, with rates especially high in the Mountain West. West Virginia and Kentucky also show regions of high suicide mortality. Patterns are broadly similar for men and women. Over time, there is a pervasive rise in suicide mortality in the working ages across all regions of the country.

**Table 1:** Population-weighted summary statistics for suicide mortality and contextual characteristics across all commuting zones, 1999–2001 and 2015–2017 (standard deviation in parentheses)

	1999–2001	2015–2017
Mortality rate per 100 k (non-Hispanic white, ages 20–64)	13.9 (5.7)	19.9 (7.0)
Male	23.4 (10.5)	31.8 (12.0)
Female	4.5 (3.4)	8.1 (5.0)
Mortality rate per 100 k (non-Hispanic white, ages 65+)	14.1 (7.6)	18.5 (7.5)
Male	23.7 (14.3)	29.3 (12.8)
Female	4.6 (5.1)	7.8 (5.6)
Percent unemployed	4.8 (1.3)	4.1 (0.9)
Percent of employment in manufacturing sector	10.9 (5.1)	7.5 (4.1)
Percent of Non-Hispanic White population ages 25+ with a bachelor’s degree or higher^a^	25.9 (7.9)	32.4 (9.0)
Percent of the population ages 15+ married	54.9 (3.4)	48.8 (3.4)
Total adherents to any religious denomination as percent of total population	50.0 (11.4)	48.3 (10.5)
Percent of households owning a firearm (state-level)	34.4 (11.8)	34.4 (11.7)
Licensed firearm sellers per 100,000	26.5 (19.4)	23.5 (18.5)
Score of restrictive gun laws (state-level)	0.9 (0.9)	0.9 (0.8)
Annual opioid prescriptions per 100,000	77.7 (27.7)	62.5 (21.4)

Source: Vital Statistics and Census data. See Supplementary Table 1 for source data for contextual characteristics

^a^Refers to both sexes combined

Table 1 also shows values of the contextual variables hypothesized to be related to suicide mortality in the two periods. The percentage of the labor force working in manufacturing industries declined from 10.9% in 2001 to 7.5% in 2017. Unemployment levels are similar at the beginning and end of the period, having risen in the interim to a peak of 10.0% in October 2009 [7].

**Fig. 2:**
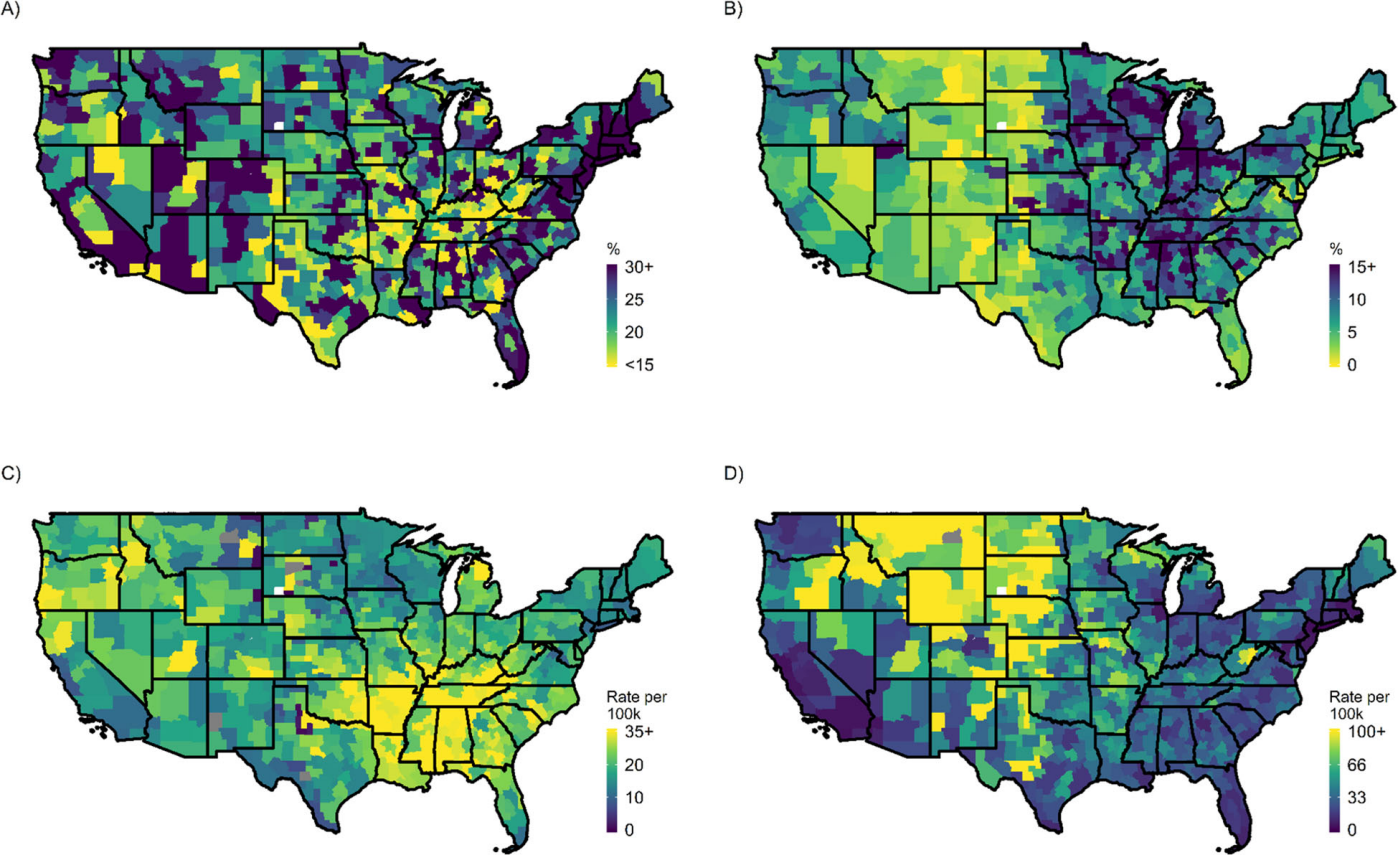
Select characteristics for commuting zones, 2017: (**a**) Age-standardized rate of college completion in the population ages 25+, (**b**) proportion of employment in the manufacturing sector, (**c**) annual opioid prescriptions per 100,000, (**d**) the number of licensed gun sellers per 100,000 (**d**). All shapefiles were obtained from publicly available Census data using the *tigris* R package [55]

Educational attainment rose over the period, the proportion married declined, and religious membership remained roughly flat. Figure 2 shows the spatial pattern of several contextual factors that vary dramatically across commuting zones. Manufacturing employment is highest in the Midwest and Central time zone and lowest in the Mountain time zone. Figure 2 shows that firearm sellers are most abundant in the Mountain time zone and Eastern Oregon. Attainment of a college degree is most prevalent on the coasts as well as in Michigan/Illinois and Colorado. The frequency of opiate prescriptions per capita is high in the East South Central region but other areas of high prescription frequency are also scattered about the country.

Table 2 presents the rate ratios from four negative binomial regression models predicting suicide mortality rates for men aged 20–64. The first model includes age in 5-year wide intervals and period (2015–2017, compared to the reference period 1999–2001). The age-pattern of mortality in mid-life is relatively flat for men, while the coefficient indicating the period 2015–2017 is 1.46, suggesting a 46% (95% confidence interval: 43–49%) increase in age-standardized suicide rates since 1999–2001. The remaining rate ratios in Models 2–4 pertain to variables expressed in standard deviation units. The second model introduces percent in manufacturing and percent unemployed. Both variables are statistically significant in the expected direction but show relatively weak associations: a one-standard deviation increase in areal unemployment increases suicide mortality by 6% (4–7%), while a one-standard-deviation unit increase in manufacturing employment reduces suicide by 2% (1–3%). The third model adds college attainment, religious participation, and proportion married. College attainment and religious participation are associated with lower suicide mortality, as expected. Contrary to expectation, the proportion married is positively associated with suicide.

**Table 2:** Rate ratios (and 95% confidence intervals) for models predicting suicide mortality by commuting zone in 1999–2001 and 2015–2017 for the non-Hispanic white male population, ages 20–64

	Model 1	Model 2	Model 3	Model 4
Intercept	0.00 (0.00–0.00)***	0.00 (0.00–0.00)***	0.00 (0.00–0.00)***	0.00 (0.00–0.00)***
Age 25–29	1.07 (1.03–1.12)***	1.07 (1.03–1.12)***	1.07 (1.03–1.11)***	1.07 (1.03–1.11)***
Age 30–34	1.11 (1.07–1.15)***	1.11 (1.07–1.15)***	1.10 (1.06–1.14)***	1.10 (1.06–1.14)***
Age 35–39	1.17 (1.13–1.22)***	1.17 (1.13–1.21)***	1.16 (1.12–1.20)***	1.16 (1.12–1.20)***
Age 40–44	1.19 (1.15–1.24)***	1.19 (1.15–1.24)***	1.19 (1.14–1.23)***	1.19 (1.15–1.23)***
Age 45–49	1.19 (1.15–1.24)***	1.19 (1.14–1.24)***	1.19 (1.15–1.23)***	1.19 (1.15–1.23)***
Age 50–54	1.15 (1.10–1.19)***	1.14 (1.10–1.19)***	1.14 (1.10–1.18)***	1.15 (1.11–1.19)***
Age 55–59	1.11 (1.06–1.15)***	1.10 (1.06–1.15)***	1.11 (1.07–1.15)***	1.11 (1.07–1.15)***
Age 60–64	0.96 (0.92–1.00)*	0.95 (0.92–0.99)*	0.96 (0.93–1.00)*	0.97 (0.93–1.00)
2017	1.46 (1.43–1.49)***	1.48 (1.45–1.51)***	1.58 (1.54–1.61)***	1.56 (1.52–1.60)***
Unemployment		1.06 (1.04–1.07)***	0.97 (0.96–0.99)***	0.97 (0.96–0.99)***
Manufacturing		0.98 (0.97–0.99)***	0.93 (0.92–0.94)***	0.93 (0.92–0.95)***
College			0.88 (0.87–0.89)***	0.93 (0.92–0.95)***
Marriage			1.05 (1.03–1.06)***	1.02 (1.01–1.04)*
Religion			0.96 (0.95–0.97)***	0.97 (0.96–0.98)***
Gun sellers				1.08 (1.06–1.10)***
Prescribing				1.10 (1.09–1.12)***
Over-dispersion	***	***	***	***
AIC	51,505	51,412	50,786	50,447
R2	0.4	0.37	0.81	0.87
N	12,240	12,240	12,240	12,240

*Unemployment* Percent of labor force unemployed, *Manufacturing* Percent of employment in manufacturing, *College* Percent of NHW population 25+ with a college degree, *Marriage* Percent of NHW population ages 15+ married, *Religion* Percent of total population belonging to any religious denomination, *Gun sellers* Ratio of total licensed firearm sellers to total population, *Prescribing* Ratio of total annual opioid prescriptions to total population

* *p* < 0.05; *** *p* < 0.001

The final model adds two variables related to access to lethal means: the prevalence of gun sellers and the frequency of opiate prescriptions. Both show strong predictive power. A one-standard-deviation increase in opiate prescribing increases suicide mortality by 10% (9–12%) while an increase in gun sellers raises mortality by 8% (6–10%). When all variables are present in Model 4, an increase in manufacturing percentage is associated with a reduction of 7% (5–8%) in suicide mortality, a larger reduction than when it is first introduced. College attainment and religious participation retain their expected direction, while unemployment and marriage prevalence are not in the hypothesized direction but one-standard deviation changes in their values affect predicted levels of suicide by only 2–3%.

The successive introduction of ecological variables related to both socioeconomic vulnerability and access to lethal means has little effect on the coefficient for 2015–2017. When the only other variables in the model are age groups, observations in 2017 are associated with a 46% (43–49%) higher suicide death rate. When all seven ecological variables are added, observations in 2017 are associated with a 56% (52–60%) higher death rate. For working age men, we have not succeeded in accounting for the pervasive, national-level rise in suicide mortality between 1999 and 2001 and 2015–2017.

Tables 3, 4 and 5 present rate ratios for these same models when applied to data for women aged 20–64, men age 65+, and women age 65+. Below, we summarize results for Model 4 when all ecological variables are present:

- The percent in manufacturing is associated with reductions in suicide mortality that range from 7% for males in both age groups to 12% for working age women and 16% for older women. The association is always in the expected direction.
- The percent unemployed has low predictive power ranging from − 4 to + 1%.
- The percent completing college is not a significant predictor of suicide for older women, but reduces predicted levels by 5–7% for other groups.
- The percent married is not significantly associated with mortality for working age people but a one-standard deviation increase in marriage reduces suicide mortality by 5% (2–8%) for people above age 65.
- A higher degree of religious participation reduces suicide mortality for all groups. For working age men and women the reduction is 3–4% but for older persons the reduction is 12% (10–13%) for men and 17% (14–20%) for women. It is one of the most potent ecological predictors of suicide for older persons.
- The prevalence of gun sellers is associated with increased suicide among women 20–64 (9%, 1–16%) but not older women. However, it is powerfully associated among men of all age groups. A one-standard deviation increase in the prevalence of gun sellers increases mortality among working-age men by 8% (6–10%) and by 15% (12–19%) among men aged 65 + .
- Opiate prescribing patterns have substantial predictive power for suicide among all groups. The increase in suicide rates for a one-standard deviation increase in per capita opiate prescriptions ranges from 7% (2–12%) for older women to 13% (11–15%) for younger women.

**Table 3:** Rate (and 95% confidence intervals) ratios for models predicting suicide mortality by commuting zone in 1999–2001 and 2015–2017 for the non-Hispanic white female population, ages 20–64

	Model 1	Model 2	Model 3	Model 4
Intercept	0.00 (0.00–0.00)***	0.00 (0.00–0.00)***	0.00 (0.00–0.00)***	0.00 (0.00–0.00)***
Age 25–29	1.27 (1.18–1.37)***	1.27 (1.18–1.36)***	1.26 (1.18–1.36)***	1.26 (1.17–1.35)***
Age 30–34	1.62 (1.51–1.74)***	1.62 (1.51–1.74)***	1.61 (1.50–1.72)***	1.60 (1.49–1.71)***
Age 35–39	1.93 (1.81–2.07)***	1.93 (1.80–2.07)***	1.90 (1.78–2.03)***	1.90 (1.78–2.03)***
Age 40–44	2.19 (2.04–2.34)***	2.19 (2.05–2.34)***	2.16 (2.02–2.30)***	2.15 (2.01–2.29)***
Age 45–49	2.23 (2.08–2.38)***	2.23 (2.08–2.38)***	2.20 (2.06–2.34)***	2.19 (2.05–2.34)***
Age 50–54	2.23 (2.09–2.38)***	2.23 (2.08–2.38)***	2.20 (2.06–2.35)***	2.20 (2.06–2.35)***
Age 55–59	2.01 (1.88–2.15)***	2.01 (1.87–2.15)***	1.98 (1.86–2.12)***	1.98 (1.86–2.12)***
Age 60–64	1.59 (1.48–1.70)***	1.58 (1.47–1.70)***	1.57 (1.46–1.68)***	1.57 (1.47–1.68)***
2017	1.74 (1.69–1.79)***	1.70 (1.64–1.75)***	1.70 (1.64–1.78)***	1.72 (1.65–1.80)***
Unemployment		1.05 (1.03–1.07)***	0.97 (0.95–1.00)*	0.96 (0.94–0.99)**
Manufacturing		0.92 (0.90–0.93)***	0.88 (0.86–0.90)***	0.88 (0.86–0.90)***
College			0.90 (0.89–0.92)***	0.95 (0.93–0.97)***
Marriage			1.00 (0.97–1.02)	0.99 (0.96–1.02)
Religion			0.95 (0.93–0.97)***	0.96 (0.94–0.97)***
Gun sellers				1.02 (0.98–1.06)
Prescribing				1.13 (1.11–1.15)***
Over-dispersion	***	***	***	***
AIC	32,867	32,757	32,625	32,482
R2	0.66	0.6	0.8	0.83
N	12,240	12,240	12,240	12,240

*Unemployment* Percent of labor force unemployed, *Manufacturing* Percent of employment in manufacturing, *College* Percent of NHW population 25+ with a college degree, *Marriage* Percent of NHW population ages 15+ married, *Religion* Percent of total population belonging to any religious denomination, *Gun sellers* Ratio of total licensed firearm sellers to total population, *Prescribing* Ratio of total annual opioid prescriptions to total population

* *p* < 0.05; ** *p* < 0.01; *** *p* < 0.001

**Table 4:** Rate ratios (and 95% confidence intervals) for models predicting suicide mortality by commuting zone in 1999–2001 and 2015–2017 for the non-Hispanic white male population, ages 65+

	Model 1	Model 2	Model 3	Model 4
Intercept	0.00 (0.00–0.00)***	0.00 (0.00–0.00)***	0.00 (0.00–0.00)***	0.00 (0.00–0.00)***
Age 70–74	1.17 (1.11–1.22)***	1.17 (1.11–1.22)***	1.16 (1.11–1.21)***	1.16 (1.11–1.21)***
Age 75–79	1.44 (1.38–1.52)***	1.44 (1.37–1.51)***	1.44 (1.37–1.50)***	1.44 (1.38–1.50)***
Age 80–84	1.75 (1.66–1.84)***	1.75 (1.66–1.84)***	1.75 (1.67–1.83)***	1.75 (1.67–1.84)***
Age 85–89	2.18 (2.08–2.30)***	2.18 (2.08–2.30)***	2.19 (2.09–2.29)***	2.21 (2.11–2.31)***
2017	1.05 (1.02–1.08)**	1.06 (1.02–1.10)***	1.08 (1.03–1.12)***	1.04 (1.00–1.09)
Unemployment		1.09 (1.08–1.11)***	1.00 (0.98–1.02)	1.01 (0.99–1.03)
Manufacturing		0.94 (0.92–0.96)***	0.91 (0.89–0.93)***	0.93 (0.91–0.94)***
College			0.88 (0.86–0.89)***	0.95 (0.93–0.97)***
Marriage			0.99 (0.97–1.02)	0.95 (0.92–0.98)***
Religion			0.88 (0.86–0.90)***	0.88 (0.87–0.90)***
Gun sellers				1.15 (1.12–1.19)***
Prescribing				1.11 (1.09–1.13)***
Over-dispersion	***	***	***	***
AIC	24,222	24,090	23,811	23,638
R2	0.72	0.69	0.85	0.78
N	6800	6800	6800	6800

*Unemployment* Percent of labor force unemployed, *Manufacturing* Percent of employment in manufacturing, *College* Percent of NHW population 25+ with a college degree, *Marriage* Percent of NHW population ages 15+ married, *Religion* Percent of total population belonging to any religious denomination, *Gun sellers* Ratio of total licensed firearm sellers to total population, *Prescribing* Ratio of total annual opioid prescriptions to total population

** *p* < 0.01; *** *p* < 0.001

**Table 5:** Rate ratios (and 95% confidence intervals) for models predicting suicide mortality by commuting zone in 1999–2001 and 2015–2017 for the non-Hispanic white female population, ages 65+

	Model 1	Model 2	Model 3	Model 4
Intercept	0.00 (0.00–0.00)***	0.00 (0.00–0.00)***	0.00 (0.00–0.00)***	0.00 (0.00–0.00)***
Age 70–74	0.87 (0.80–0.95)**	0.87 (0.80–0.94)***	0.87 (0.80–0.94)***	0.87 (0.80–0.94)***
Age 75–79	0.80 (0.73–0.87)***	0.80 (0.73–0.87)***	0.80 (0.73–0.87)***	0.80 (0.73–0.87)***
Age 80–84	0.70 (0.63–0.77)***	0.69 (0.63–0.77)***	0.70 (0.63–0.77)***	0.70 (0.63–0.77)***
Age 85–89	0.63 (0.57–0.69)***	0.62 (0.57–0.69)***	0.63 (0.57–0.69)***	0.63 (0.57–0.69)***
2017	1.31 (1.23–1.40)***	1.19 (1.12–1.28)***	1.11 (1.02–1.21)*	1.15 (1.05–1.26)**
Unemployment		1.03 (0.99–1.07)	1.01 (0.96–1.06)	1.00 (0.95–1.05)
Manufacturing		0.81 (0.77–0.84)***	0.85 (0.81–0.89)***	0.84 (0.80–0.88)***
College			1.00 (0.96–1.03)	1.00 (0.96–1.05)
Marriage			0.93 (0.88–0.99)*	0.95 (0.90–1.02)
Religion			0.82 (0.79–0.86)***	0.83 (0.80–0.86)***
Gun sellers				0.91 (0.84–0.99)*
Prescribing				1.07 (1.02–1.12)**
Over-dispersion	***	***	***	***
AIC	11,180	11,069	10,983	10,974
R2	0.7	0.74	0.79	0.78
N	6800	6800	6800	6800

*Unemployment* Percent of labor force unemployed, *Manufacturing* Percent of employment in manufacturing, *College* Percent of NHW population 25+ with a college degree, *Marriage* Percent of NHW population ages 15+ married, *Religion* Percent of total population belonging to any religious denomination, *Gun sellers* Ratio of total licensed firearm sellers to total population, *Prescribing* Ratio of total annual opioid prescriptions to total population

* *p* < 0.05; ** *p* < 0.01; *** *p* < 0.001

The introduction of ecological variables does not account for any part of the rise in suicide mortality for three of four groups between 1999 and 2017. The only exception is older women, for whom the rise of 31% (23–40%) in suicide mortality that is estimated when only age is present in Model 1 is reduced to 15% (5–26%) when all seven ecological variables are included. Most of the reduction is attributable to the introduction of percent in manufacturing and unemployment in Model 2.

As a sensitivity analysis, we include deaths of undetermined intent (ICD-10 Y10-Y34) with confirmed suicides (Supplementary Tables 2, 3, 4 and 5). These codes are applied at different rates across the United States and may be capturing misclassified suicide deaths. We find that some of the contextual characteristics related to vulnerability are sensitive to including these additional deaths. Unemployment for men 20–64 and college attainment for women 20–64 are not statistically significant when including deaths of undetermined intent. All other estimates are substantively unaffected.

## Discussion

The principal hypothesis motivating this study is that the proportion of the labor force in manufacturing industries in an area is negatively associated with suicide mortality. This hypothesis is supported by results based on 704 commuting zones. Male suicide mortality is reduced by 7% for each standard deviation increase in the proportion in manufacturing. Perhaps surprisingly, female suicide mortality is more strongly affected by the manufacturing proportion than is male mortality. Women aged 20–64 have reductions of 12% and women aged 65+ by 16% for each standard deviation unit increase in the manufacturing percentage. In a related study, Charles et al. [16] found that a decline in manufacturing percentage in a commuting zone was significantly associated with a rise in mortality from opioid overdose between 2000 and 2016.

Few other studies have investigated the relation between suicide and economic circumstances separately for different age/sex groups. Lin and Chen [39] found that the suicide mortality of older people (aged 55–64 in their analysis) was more responsive to the economic cycle than that of younger people. One recent study found that women’s mortality from suicide reacted more strongly to economic recession than men’s [11]. A time-series study of suicide mortality in England and Wales, 1962–96, separately investigated men and women aged 25–34 and 60+ (Gunnell et al. 2003) [26]. It found that male unemployment was significantly associated (and positively) only with the mortality of older males and that GDP was significantly associated (and negatively) only with the mortality of older females. One might speculate that older women’s suicide is the most responsive to economic conditions because their children may leave their area when economic conditions are poor, or their husbands may be more likely to die, or their pensions may be adversely affected. This result deserves additional study.

We find that the level of unemployment in an area is only weakly connected to suicide mortality and not always in the expected direction. This result is similar to that of Trgovac et al. [52], who used a spatial error regression model on county data during 2000–06 and found county unemployment levels to be an insignificant predictor of working-age male suicide mortality. It is possible that these results reflect the fact that our observations are drawn from two periods of very low unemployment and miss the large increase associated with the Great Recession. On the other hand, Harper and Bruckner’s [30] time series analysis of suicide mortality finds no effect of the Great Recession.

We have differentiated between the factors that may incline an individual to attempt suicide and the means available to do so. We investigate two areal characteristics that are related to the means for killing oneself, the number of gun sellers per capita and the per capita number of opioid prescriptions per capita. Both are strongly related to death rates from suicide. Among men, a one-standard deviation increase in the prevalence of gun sellers increases mortality by 8% among working-age men and by 15% among men aged 65+. Among women, the frequency of gun sellers is insignificantly related to suicide mortality at age 65+ or raises mortality by only 2% at ages 20–64. This result mirrors sex differences in the proportion of suicide deaths from the use of guns; men represent 86% of firearm suicide victims [15].

A one-standard deviation increase in opiate prescriptions per capita raises suicide mortality in the working ages by 10% for men and 13% for women; for people 65+, the increase is 11% for men and 7% for women. This variable is among the strongest predictors of suicide across the board. While these drugs may have some effect in reducing the prevalence of pain and pain-associated suicide, it is clear that the net effect of additional prescriptions is to raise suicide rates. This connection is likely a combination of two factors: the direct use of opiates as a lethal means for suicide, but also the manner in which the aggressive marketing and the scale-up of prescribing was specifically targeted to the most vulnerable populations in the country [20, 40, 47, 50]. In this way, our measure of prescription rates may be serving as a proxy for other unmeasured forms of contextual vulnerability to suicide.

The remaining variables (college completion, marriage prevalence, and religious participation) are treated as controls in the analysis. They have a modest effect on suicide, mostly in the expected direction. The one variable with stronger effects is religious participation among older individuals, which reduces suicide by 12% for men and 17% for women.

The strongest contextual predictors of suicide mortality are the manufacturing percentage and opiate prescriptions for all age/sex groups, gun accessibility for men, and religious participation for older people. With one exception, these and other variables are not successful in accounting for rising suicide mortality over the period 1999–2001 to 2015–17. The exception pertains to women 65+, for whom the introduction of manufacturing (and unemployment) into a model containing only age reduces the increase in suicide mortality over this period from 31 to 19%. The manufacturing percentage in an area is not only predictive of cross-sectional suicide mortality differentials among older women but helps to account for suicide increase among this group as well.

One weakness of our analysis of trends is that one of our variables, gun sellers per capita, only refers to 2017, so that changes in its value could not account for trends. As noted earlier, we have investigated two other variables related to gun availability measured at the state-level. The prevalence of guns in households in one’s state of residence in 2004 is cross-sectional. The other variable considered relates to the restrictiveness of gun laws in one’s state of residence and its values vary over the period of observation (Supplementary Table 1), but these variables were less predictive than gun sellers in supplemental analyses (results available upon request).

Another limitation of our analyses is that it is based on aggregate-level data at the level of the commuting zone and thus the results cannot be generalized to individuals. At the same time, we avoid possible confounding between employment and suicide risk by unmeasured individual-level attributes when using individual-level data alone. Ideally, one would combine both individual- and contextual-level characteristics, which would allow for a more comprehensive assessment of the joint influence of the broader social context and individual characteristics and their possible interactions.

We should also note that it is possible that the coding of suicide may vary across the country related differential training of death certificate certifiers, the number of autopsies and other evaluations, access to medical records, and social pressures to avoid the stigma of suicide. In addition, for some proportion of violent deaths the intent cannot always be determined [25], which may be particularly true for deaths from opioid-overdose [44]. We conducted a sensitivity analysis including ICD-10 codes Y10-Y34, but there may still be other unobserved patterns of differential coding that vary along important demographic or geographic characteristics [12, 17].

It is possible that spatial autocorrelation exists across commuting zone suicide death rates (i.e. observations are not independent and identically distributed, or *iid*, conditional on observed covariates, but rather are more correlated with nearer observations than those further away). In this case, conventional regression models that treat observations as *iid* may produce biased coefficient estimates and inappropriately narrow standard errors. In additional analyses, we tested fitting a Bayesian hierarchical model with a spatially correlated error term following the *Besag*-*York*-Mollié (BYM) specification to account for potential spatial autocorrelation in observed death rates [4, 6]. Our substantive findings regarding access to lethal means (gun sellers and prescribing rates) are largely robust to accounting for spatial autocorrelation, but the associations with manufacturing and unemployment are more sensitive, especially for men. This is perhaps due to spatial autocorrelation in the measures of manufacturing and unemployment themselves, but raises the possibility that this variation might be due to some unobserved, underlying common cause that is spatially structured.

## Conclusions

Failure to account for the dramatic rise in suicide mortality across all commuting zones in the United States over this period is a critical finding from our analysis. Despite a relative lack of comprehensive analyses on the correlates of suicide mortality over the past two decades, popular hypotheses for the rise in suicide mortality often cite the measures of socioeconomic depression and access to lethal means that we have analyzed here. While our models demonstrate that these factors are indeed consistently related to suicide mortality in the expected directions and explain a large proportion of the variation in suicide mortality across commuting zones, changes therein cannot alone explain changes in suicide mortality between 1999 and 2001 and 2015–2017. Even so, our analyses suggest that access to lethal means is as an influential predictor of suicide mortality as contextual vulnerability due to employment conditions. These factors, gun availability and opioid prescriptions, are also much more straightforward to influence via public policy, such as implementing more restrictive gun laws and the effective regulation of predatory pharmaceutical marketing.

## Supplementary information

**Additional file 1: Supplementary material.** Information on data sources for mortality data and all contextual characteristics. Sensitivity analyses including deaths of undetermined intent (ICD-10 codes Y10-Y34).

## Data Availability

The data that support the findings of this study are available from the National Center for Health Statistics but restrictions apply to the availability of these data, which were used under license for the current study, and so are not publicly available. All contextual data are available from public repositories reported in the manuscript. All code for analysis will be available at https://github.com/ngraetz/nhw_suicide.

## Abbreviations

CZ: Commuting zone
USDA: United States Department of Agriculture
WHO: World Health Organization
GDP: Gross domestic product
CDC: Centers for Disease Control and Prevention
US: United States
