# Supplementary material for: Ecological factors associated with suicide mortality among non-Hispanic whites
Source: BMC Public Health. 2020 Sep 3;20:1339. doi: 10.1186/s12889-020-09379-w (PMC7469302; doi:10.1186/s12889-020-09379-w)
Supplement: Supplementary file 1 — Additional file 1: Supplementary material. Information on data sources for mortality data and all contextual characteristics. Sensitivity analyses including deaths of undetermined intent (ICD-10 codes Y10-Y34). [file 12889_2020_9379_MOESM1_ESM.docx]

**Supplementary Table 1.** Sources for mortality data and all contextual characteristics.

|  | Source | Years | Notes |
| --- | --- | --- | --- |
| Percent of labor force unemployed | Bureau of Economic Affairs (BEA) | 2001, 2017 | 2001 values used for 2000. |
| Percent of employment in manufacturing | Bureau of Economic Affairs (BEA) | 2001, 2017 | 2001 values used for 2000. |
| Percent of sex-specific Non-Hispanic White population ages 25+ with a bachelor’s degree or higher | Census/American Community Survey (ACS) | 2000, 2015-2019 | 5-year pooled ACS data used for 2017. |
| Percent of the population ages 15+ married | Census/American Community Survey (ACS) | 2000, 2015-2019 | 5-year pooled ACS data used for 2017. |
| Total adherents to any religious denomination as percent of total population | U.S. Religion Census: Longitudinal Religious Congregations and Membership | 2000, 2010 | 2010 values used for 2017. |
| Percent of households owning a firearm | The Behavioral Risk Factor Surveillance System | 2004 | State-level data. 2004 values used for 2000 and 2017. |
| Gun laws | Rand State Firearm Law Database | 2000, 2017 | State-level data. Includes handgun restrictions, long-gun restrictions, background checks. |
| Gun sellers | Bureau of Alcohol, Tobacco, Firearms and Explosives (ATF) | 2017 | 2017 value used for 2000. Includes all licensed firearm importers, dealers and pawnbrokers. |
| Annual opioid prescriptions per 1000 population | Centers for Disease Control and Prevention (CDC) | 2006, 2017 | 2006 values used for 2000 |

**Supplementary Table 2.** Rate ratios (and 95% confidence intervals) for models predicting suicide mortality by commuting zone in 1999-2001 and 2015-2017 for the non-Hispanic white male population, ages 20-64. Includes deaths of undetermined intent (ICD-10 codes Y10-Y34).

|  | Model 1 | | Model 2 | | Model 3 | | Model 4 | |
| --- | --- | --- | --- | --- | --- | --- | --- | --- |
| Intercept | 0.00 | *** | 0.00 | *** | 0.00 | *** | 0.00 | *** |
| Age 25 | 1.09 | *** | 1.09 | *** | 1.09 | *** | 1.09 | *** |
| Age 30 | 1.14 | *** | 1.14 | *** | 1.13 | *** | 1.13 | *** |
| Age 35 | 1.21 | *** | 1.21 | *** | 1.20 | *** | 1.20 | *** |
| Age 40 | 1.22 | *** | 1.22 | *** | 1.21 | *** | 1.21 | *** |
| Age 45 | 1.21 | *** | 1.20 | *** | 1.20 | *** | 1.20 | *** |
| Age 50 | 1.14 | *** | 1.14 | *** | 1.13 | *** | 1.13 | *** |
| Age 55 | 1.09 | *** | 1.08 | *** | 1.08 | *** | 1.09 | *** |
| Age 60 | 0.93 | *** | 0.92 | *** | 0.93 | *** | 0.93 | *** |
| 2017 | 1.46 | *** | 1.48 | *** | 1.55 | *** | 1.55 | *** |
| Unemployment |  |  | 1.06 | *** | 1.00 |  | 0.99 |  |
| Manufacturing |  |  | 0.97 | *** | 0.92 | *** | 0.93 | *** |
| College |  |  |  |  | 0.91 | *** | 0.96 | *** |
| Marriage |  |  |  |  | 1.04 | *** | 1.02 | * |
| Religion |  |  |  |  | 0.97 | *** | 0.98 | *** |
| Gun sellers |  |  |  |  |  |  | 1.07 | *** |
| Prescribing |  |  |  |  |  |  | 1.11 | *** |
| Over-dispersion | *** |  | *** |  | *** |  | *** |  |
| AIC | 52920 |  | 52797 |  | 52419 |  | 52071 |  |
| R2 | 0.29 |  | 0.24 |  | 0.6 |  | 0.71 |  |
| N | 12240 |  | 12240 |  | 12240 |  | 12240 |  |
| * p < 0.05; ** p < 0.01; *** p < 0.001 | | | | | | | | |

**Supplementary Table 3.** Rate ratios (and 95% confidence intervals) for models predicting suicide mortality by commuting zone in 1999-2001 and 2015-2017 for the non-Hispanic white female population, ages 20-64. Includes deaths of undetermined intent (ICD-10 codes Y10-Y34).

|  | Model 1 | | Model 2 | | Model 3 | | Model 4 | |
| --- | --- | --- | --- | --- | --- | --- | --- | --- |
| Intercept | 0.00 | *** | 0.00 | *** | 0.00 | *** | 0.00 | *** |
| Age 25 | 1.32 | *** | 1.32 | *** | 1.32 | *** | 1.31 | *** |
| Age 30 | 1.70 | *** | 1.70 | *** | 1.69 | *** | 1.68 | *** |
| Age 35 | 2.03 | *** | 2.03 | *** | 2.01 | *** | 2.00 | *** |
| Age 40 | 2.23 | *** | 2.23 | *** | 2.21 | *** | 2.20 | *** |
| Age 45 | 2.26 | *** | 2.26 | *** | 2.24 | *** | 2.23 | *** |
| Age 50 | 2.23 | *** | 2.22 | *** | 2.20 | *** | 2.20 | *** |
| Age 55 | 1.95 | *** | 1.95 | *** | 1.93 | *** | 1.93 | *** |
| Age 60 | 1.51 | *** | 1.50 | *** | 1.50 | *** | 1.50 | *** |
| 2017 | 1.76 | *** | 1.72 | *** | 1.72 | *** | 1.74 | *** |
| Unemployment |  |  | 1.06 | *** | 1.01 |  | 1.00 |  |
| Manufacturing |  |  | 0.91 | *** | 0.89 | *** | 0.89 | *** |
| College |  |  |  |  | 0.94 | *** | 1.00 |  |
| Marriage |  |  |  |  | 1.00 |  | 0.99 |  |
| Religion |  |  |  |  | 0.96 | *** | 0.97 | ** |
| Gun sellers |  |  |  |  |  |  | 1.03 |  |
| Prescribing |  |  |  |  |  |  | 1.15 | *** |
| Over-dispersion | *** |  | *** |  | *** |  | *** |  |
| AIC | 35253 |  | 35115 |  | 35056 |  | 34853 |  |
| R2 | 0.47 |  | 0.39 |  | 0.55 |  | 0.66 |  |
| N | 12240 |  | 12240 |  | 12240 |  | 12240 |  |
| * p < 0.05; ** p < 0.01; *** p < 0.001 | | | | | | | | |

**Supplementary Table 4.** Rate ratios (and 95% confidence intervals) for models predicting suicide mortality by commuting zone in 1999-2001 and 2015-2017 for the non-Hispanic white male population, ages 65+. Includes deaths of undetermined intent (ICD-10 codes Y10-Y34).

|  | Model 1 | | Model 2 | | Model 3 | | Model 4 | |
| --- | --- | --- | --- | --- | --- | --- | --- | --- |
| Intercept | 0.00 | *** | 0.00 | *** | 0.00 | *** | 0.00 | *** |
| Age 70 | 1.15 | *** | 1.15 | *** | 1.15 | *** | 1.15 | *** |
| Age 75 | 1.43 | *** | 1.43 | *** | 1.42 | *** | 1.42 | *** |
| Age 80 | 1.73 | *** | 1.73 | *** | 1.73 | *** | 1.73 | *** |
| Age 85 | 2.15 | *** | 2.16 | *** | 2.16 | *** | 2.18 | *** |
| 2017 | 1.05 | ** | 1.07 | *** | 1.08 | *** | 1.05 | * |
| Unemployment |  |  | 1.09 | *** | 1.00 |  | 1.01 |  |
| Manufacturing |  |  | 0.94 | *** | 0.91 | *** | 0.93 | *** |
| College |  |  |  |  | 0.88 | *** | 0.95 | *** |
| Marriage |  |  |  |  | 0.99 |  | 0.95 | *** |
| Religion |  |  |  |  | 0.88 | *** | 0.89 | *** |
| Gun sellers |  |  |  |  |  |  | 1.16 | *** |
| Prescribing |  |  |  |  |  |  | 1.11 | *** |
| Over-dispersion | *** |  | *** |  | *** |  | *** |  |
| AIC | 24402 |  | 24275 |  | 23996 |  | 23813 |  |
| R2 | 0.73 |  | 0.69 |  | 0.86 |  | 0.79 |  |
| N | 6800 |  | 6800 |  | 6800 |  | 6800 |  |
| * p < 0.05; ** p < 0.01; *** p < 0.001 | | | | | | | | |

**Supplementary Table 5.** Rate ratios (and 95% confidence intervals) for models predicting suicide mortality by commuting zone in 1999-2001 and 2015-2017 for the non-Hispanic white female population, ages 65+. Includes deaths of undetermined intent (ICD-10 codes Y10-Y34).

|  | Model 1 | | Model 2 | | Model 3 | | Model 4 | |
| --- | --- | --- | --- | --- | --- | --- | --- | --- |
| Intercept | 0.00 | *** | 0.00 | *** | 0.00 | *** | 0.00 | *** |
| Age 70 | 0.87 | *** | 0.86 | *** | 0.86 | *** | 0.86 | *** |
| Age 75 | 0.79 | *** | 0.78 | *** | 0.78 | *** | 0.78 | *** |
| Age 80 | 0.71 | *** | 0.71 | *** | 0.71 | *** | 0.71 | *** |
| Age 85 | 0.66 | *** | 0.66 | *** | 0.66 | *** | 0.66 | *** |
| 2017 | 1.33 | *** | 1.22 | *** | 1.15 | *** | 1.18 | *** |
| Unemployment |  |  | 1.03 |  | 1.01 |  | 1.01 |  |
| Manufacturing |  |  | 0.81 | *** | 0.85 | *** | 0.85 | *** |
| College |  |  |  |  | 0.99 |  | 1.01 |  |
| Marriage |  |  |  |  | 0.95 |  | 0.96 |  |
| Religion |  |  |  |  | 0.83 | *** | 0.84 | *** |
| Gun sellers |  |  |  |  |  |  | 0.93 |  |
| Prescribing |  |  |  |  |  |  | 1.06 | ** |
| Over-dispersion | *** |  | *** |  | *** |  | *** |  |
| AIC | 11758 |  | 11646 |  | 11564 |  | 11558 |  |
| R2 | 0.72 |  | 0.75 |  | 0.8 |  | 0.79 |  |
| N | 6800 |  | 6800 |  | 6800 |  | 6800 |  |
| * p < 0.05; ** p < 0.01; *** p < 0.001 | | | | | | | | |
